# Supplementary material for: FastqPuri: high-performance preprocessing of RNA-seq data
Source: BMC Bioinformatics. 2019 May 3;20:226. doi: 10.1186/s12859-019-2799-0 (PMC6500068; doi:10.1186/s12859-019-2799-0)
Supplement: Supplementary file 2 — Archive of FastqPuri. Archive containing all files needed to install and run FastqPuri v1.0.6. Date stamp March 22, 2019. (GZ 47,819 kb) [file 12859_2019_2799_MOESM2_ESM.gz › FastqPuri-1.0.6/html/globals_w.html]

FastqPuri: File Members


FastqPuri


Here is a list of all documented file members with links to the documentation:

### - w -

- write\_info()
  : stats\_info.c
  , stats\_info.h
- write\_summary\_TF()
  : io\_trimFilter.h
  , io\_trimFilter.c
- write\_summary\_TFDS()
  : io\_trimFilterDS.h


---

Generated on Mon Mar 19 2018 23:42:01 for FastqPuri by  

 1.8.14
